# Defining a critical period for inhibitory circuits within the somatosensory cortex

**DOI:** 10.1038/s41598-017-07400-8

**Published:** 2017-08-04

**Authors:** Shun Qiang Lo, Judy C. G. Sng, George J. Augustine

**Affiliations:** 1 Department of Physiology, Yong Loo Lin School of Medicine, National University of Singapore, Singapore, Singapore; 2 Lee Kong Chian School of Medicine, Nanyang Technological University, Singapore, Singapore; 3 Institute of Molecular and Cell Biology (A*STAR), Singapore, Singapore; 4 Marine Biological Laboratory, Woods Hole, USA; 5 Department of Pharmacology, Yong Loo Lin School of Medicine, National University of Singapore, Singapore, Singapore; 6 Singapore Institute of Clinical Sciences, Agency for Science and Technology (A*STAR), Singapore, Singapore

## Abstract

Although experience-dependent changes in brain inhibitory circuits are thought to play a key role during the “critical period” of brain development, the nature and timing of these changes are poorly understood. We examined the role of sensory experience in sculpting an inhibitory circuit in the primary somatosensory cortex (S1) of mice by using optogenetics to map the connections between parvalbumin (PV) expressing interneurons and layer 2/3 pyramidal cells. Unilateral whisker deprivation decreased the strength and spatial range of inhibitory input provided to pyramidal neurons by PV interneurons in layers 2/3, 4 and 5. By varying the time when sensory input was removed, we determined that the critical period closes around postnatal day 14. This yields the first precise time course of critical period plasticity for an inhibitory circuit.

## Introduction

The “critical period” is a developmental phase characterized by heightened neuronal plasticity that makes cortical circuits particularly susceptible to regulation by the sensory input provided by environmental stimuli^1^. Critical periods occur early in postnatal development and are often brief in duration^2, 3^. They were first identified in the visual cortex by Hubel and Wiesel^4^, who found that closing one eye, to deprive this eye of visual stimulation during development, caused cortical visual responses to be biased towards the open eye^4, 5^. This phenomenon is known as ocular dominance plasticity^5^ and reflects extensive synaptic reorganization that is based on the timing and type of sensory experience. Similarly, critical period plasticity occurs in the somatosensory cortex of rodents and is reflected in disorganization of the structure of the layer 4 barrel field following loss of whisker input^6^ as well as an age-dependent decrease in the ability to induce long-term potentiation of thalamocortical excitatory synapses^7^.

Recent studies in both visual and somatosensory cortices have highlighted the importance of inhibitory circuits in critical period plasticity. In the visual cortex, disruption of the gene encoding one isoform of glutamic acid decarboxylase - an enzyme involved in synthesis of the inhibitory neurotransmitter, GABA - prevents the changes in ocular dominance plasticity that normally result from sensory deprivation^8^. Pharmacological reduction of inhibition also blocks ocular dominance plasticity and delays closure of the critical period^9^. Conversely, transplantation of inhibitory neurons after critical period closure is sufficient to induce ocular dominance plasticity^10^. Presumably these effects are a result of altering the balance between excitatory and inhibitory transmission that is required for normal brain function^8, 10–15^.

Relatively little is known about the precise inhibitory circuits that are affected during critical period plasticity or the timing of their critical periods, particularly in the somatosensory cortex. We have therefore examined the effects of neonatal sensory deprivation on a circuit formed by parvalbumin (PV)-expressing interneurons within the somatosensory cortex. These PV interneurons receive excitatory input from the thalamus^16–20^, layer 4^21^ and layer 2/3 excitatory neurons, as well as inhibitory input from interneurons such as basket cells, chandelier cells and Martinotti cells^22^. PV interneurons are likely candidates for critical period plasticity because they provide inhibitory control of local excitatory circuits in the barrel column. While sensory deprivation is known to decrease the strength of the inhibitory circuit between PV interneurons and layer 4 excitatory neurons^12^, it is not known whether this circuit exhibits a defined critical period. Furthermore, it is not known whether such plasticity affects inhibitory circuits within other cortical layers.

We used optogenetic mapping^23, 24^ to examine changes in the inhibitory circuit between PV interneurons and layer 2/3 pyramidal neurons occurring during the critical period. We found that chronic sensory deprivation decreases synaptic transmission within this circuit, extending previous findings in layer 4 inhibitory circuits. Mapping indicated that this activity-dependent change in circuit function preferentially affects more distant synaptic connections. Most importantly, by varying the timing of sensory deprivation, we observed that the critical period for this plasticity extends only over the first two postnatal weeks. Our identification and quantification of these changes in inhibitory circuit function yields the first definition of the critical period for inhibitory circuit function and may help account for the timing of the layer 2/3 receptive field critical period. It also paves the way for identification of the molecular mechanisms underlying inhibitory circuit changes during the critical period.

## Materials and Methods

Patch clamp electrophysiology and optogenetic mapping were combined^23^ to identify the spatial extent, synaptic strength and connectivity of cortical circuits in mouse brain and to determine how these circuits change in response to whisker deprivation.

### Animals

All procedures were approved by the Biological Resource Centre Institutional Animal Care and Use Committee. All methods were done in accordance to relevant guidelines and regulations. To map the connections between PV interneurons and pyramidal neurons, we used double transgenic mice expressing ChR2 (H134R) specifically in PV interneurons^25^. These mice were prepared by mating *Pvalb-*ires-Cre mice (129P2-*Pvalb* ^*tm1(cre)Arbr/*^J; Jax stock number 008069) expressing Cre recombinase in PV interneurons^26^ with another transgenic line having the ChR2(H134R)-eYFP fusion gene inserted, behind a floxed stop cassette, into the *Rosa26* locus 129S-Gt(ROSA)26Sortm32(CAG-COP4*H134R/EYFP)Hze/J; Jax; stock number 012569^27^.

### Histology

Histology was used to characterize expression of eYFP-tagged ChR2 in the brains of double transgenic mice. For this purpose, adult mice were anaesthetized and euthanized with an overdose of ketamine/xylazine (10 mg/kg body weight) and transcardially perfused with 0.1 M phosphate buffer saline (PBS, pH 7.4) followed by 4% paraformaldehyde. The brain was removed and subsequently post-fixed in 4% paraformaldehyde for an hour before it was transferred to 30% sucrose in 0.1 M PBS and stored at 4 °C overnight in the fixative. The brain was then frozen and a cryostat was used to cut the tissue into 30-µm thick sections. For antibody staining, sections were washed 3 times for 5 minutes in 0.1 M PBS before blocking in 0.3%-Triton-X, 3% normal goat serum in 0.1 M PBS at room temperature for 1 hour. This was followed by overnight incubation with rabbit anti-parvalbumin antibody (Swant, 1:1000 dilution) in the blocking solution at 4 °C. The sections were then washed 3 times for 10 minutes in PBS and then incubated for 1 hour with goat anti-rabbit lgG conjugated with Alexa-Fluor 680 (Invitrogen) (1:200 dilution) in the blocking solution at room temperature. After three 10-minute washes in 0.1 M PBS, sections were mounted on glass slides in ProlongGold mounting medium (Invitrogen) for viewing. Fluorescent images were obtained with a confocal microscope (Nikon AR1-A1).

### Whisker deprivation

For sensory deprivation, all large mystacial whiskers (from rows A to E and arcs 0 to 6, and including α, β, γ and δ whiskers) were removed. At P0, P3, P7, P14 or P21, whiskers from the right cheek were first trimmed with tweezers by applying slow, steady tension to the base of the whiskers, followed by the light cauterization of whisker follicles using a hot wire tip of a custom-made cautery device. Neonates were anaesthetized by rapid cooling via indirect contact with ice, while older pups (P10 and older) were anaesthetized using 2–3% isofluorane throughout the deprivation procedure. To simulate any stimulation associated with whisker trimming, whiskers on the left cheek of pups were sham-trimmed by stroking with tweezers as a control. After cauterization, pups were placed in a warmed cage and monitored throughout post-operative recovery and were then returned to their nursing mother.

### Slice preparation

Brain slices (300 µm thick) were prepared from P28-P33 mice using procedures described elsewhere^24^. Slices were cut in ice-cold cutting solution containing (in mM); 240 Sucrose, 10 Glucose, 25 NaHCO_3_, 1.25 NaH_2_PO_4_.2H_2_0, 2.5 KCl, 0.5 CaCl_2_, 7 MgCl_2_ (320–325 mOsm) and incubated in oxygenated (95% oxygen, 5% carbon dioxide) artificial cerebral spinal fluid (ACSF) solution containing (in mM); 10 Glucose, 126 NaCl, 24 NaHCO_3_, 1 NaH_2_PO_4_.2H_2_0, 2.5 KCl, 2.5 CaCl_2_, 2 MgCl_2_ (300–310 mOsm). The brain was sectioned at 50 degrees to the midline in order to obtain across-column slices similar to the procedures detailed in Finnerty *et al*.^28, 29^.

Slices were incubated at 32 °C for 30 minutes and subsequently kept at room temperature (25 °C) for another 30 minutes prior to electrophysiological recordings. Barrels were visualized by trans-illumination of the slice and all recordings were done with slices submerged in oxygenated ACSF at room temperature (24–26 degrees Celsius).

### Optogenetic mapping of inhibitory circuits

To map the connections between PV interneurons and pyramidal neurons, we used 300-µm thick brain slices from transgenic mice expressing ChR2 (H134R) in PV interneurons (see above). Spots of violet light (4 ms, 405 nm) were scanned over the brain slice in an array of 32 by 32 pixels via a FV-1000MPE laser-scanning microscope (Olympus, USA), while simultaneous electrical recordings measured responses in presynaptic interneurons or postsynaptic pyramidal neurons^23, 24^. To determine the appropriate light power for activating PV interneurons, whole-cell current clamp recordings were made from PV interneurons and action potential generation in response to light were measured. To map PV inhibitory circuit inputs onto pyramidal cells, voltage clamp recordings were done on layer 2/3 pyramidal cells to measure inhibitory postsynaptic currents resulting from photostimulation of PV interneurons. For both types of recording a MultiClamp 700B amplifier (Molecular Devices, USA) was used. Potassium gluconate intracellular solution was used, containing (in mM); 130 K-Gluconate, 10 KOH, 10 HEPES, 4 Na_2_ATP, 0.4 Na_3_GTP, 5 EGTA, 5 Disodium Phosphocreatine, 2.5 MgCl_2_ (290–295 mOsm, pH 7.25). Patch clamp recordings were made with pipettes (4–6 MΩ) pulled with a vertical puller (Narishige, Japan). Voltage clamped cells were held at a potential of −60 mV; all membrane potential measurements were corrected for a liquid junction potential of −11.8 mV.

Optogenetic circuit mapping was done with a 10x objective with a large field of view (approximately 1.6 mm^2^), allowing us to visualize interneuron inputs across layers 1–6 and along 3–4 adjacent cortical columns. For photostimulation mapping, a small laser spot was scanned across the field of view in a pseudorandom fashion, to avoid sequential stimulation of adjacent pixels. A laser power of 160 µW (405 nm, 4 ms) was used for mapping because it was the lowest capable of reliably evoking action potential firing in PV interneurons. The area over which this laser spot elicited action potential firing was then determined and defined the optical footprint for PV interneurons. IPSCs were then recorded from postsynaptic pyramidal neurons as a measure of inhibitory synaptic transmission when presynaptic PV interneurons were photostimulated by the laser spot. Input field maps were constructed by correlating the location of each photostimulation spot in the microscope field with the amplitude of the IPSC evoked at each location within a 20 ms time window following the photostimulus. Treatment with the GABA_A_ receptor antagonist bicuculline (10 µM) was used to confirm that the outward currents observed were IPSCs. The number of presynaptic IPSC inputs was estimated by division of the input field area and the optical footprint^24^; this is a lower estimate because of possible photostimulation of multiple PV interneurons within the laser beam. Data analyses were done with a custom-made program in MATLAB. Because we measured responses in single cells within individual circuits, mean values were calculated across the number of cells, rather than number of animals; very similar mean values were obtained regardless of whether number of cells or number of animals were used to calculate the means (data not shown). In addition, it was not possible to make direct intra-animal comparisons in every case; in some animals we managed to collect data from only control or deprived sides. The non-parametric Mann-Whitney two-tailed test and two-sample Kolgomorov-Smirnov tests were used to test for the level of statistical significance at α = 0.05 for between-group changes. Linescan analyses were compared with two-way ANOVA with Bonferroni post-hoc tests. All relevant data are available from the authors.

## Results

We used whisker deprivation to define the role of sensory activity in the development of inhibitory circuits within the somatosensory cortex. This was done by removing all whiskers from the right side while keeping the other side intact, to serve as a control. This allowed us to compare the effects of sensory experience and its loss in the same animal. Changes in circuit function initially were examined in response to chronic whisker deprivation beginning at age P0. Because the light cauterization that we used removes whiskers for approximately 1 month^29^, and both excitatory and inhibitory circuits in the cortex are fully developed in mice by P28^30^, we could examine P28–P33 mice to identify long-lasting circuit changes caused by such changes in sensory experience.

### Deprivation reduces inhibition by parvalbumin interneurons

Amongst the numerous types of cortical interneurons^31^, PV interneurons are a plausible site for experience-dependent plasticity because they receive substantial sensory input in response to whisker activity during development^16–21^ and have been proposed to mediate ocular dominance plasticity in the visual cortex^10^. PV interneurons also are dominant sources of inhibition that can synchronize the electrical activity of cortical pyramidal neurons^32, 33^.

**Figure 1:**
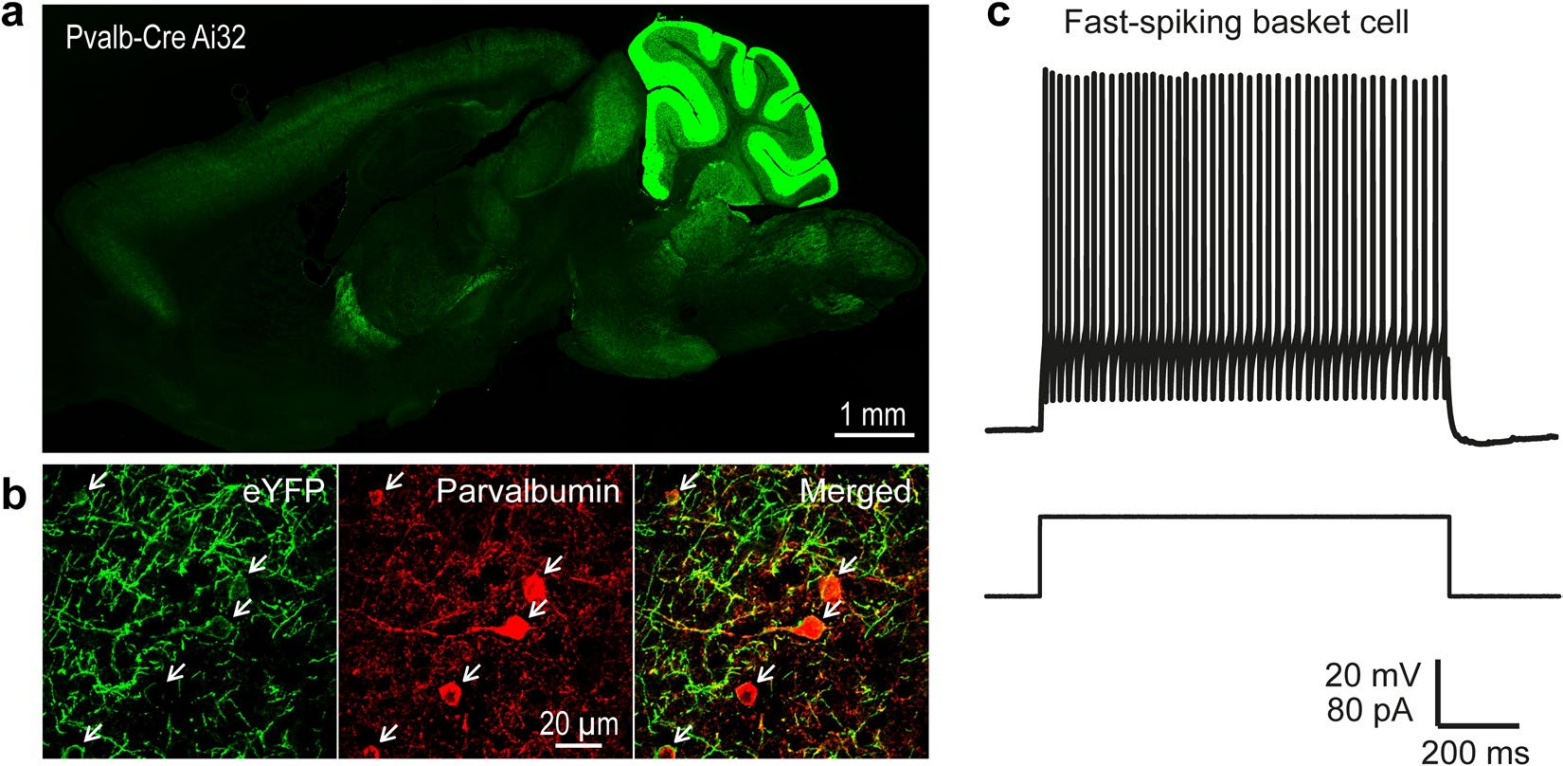
Expression of eYFP-tagged ChR2 in PV interneurons in the somatosensory cortex. (**a**) Expression of eYFP-tagged ChR2 (in green) in the cortex and cerebellum of double-transgenic Prv-Cre x Ai32 mice. (**b**) eYFP-tagged ChR2 (green) is expressed in PV interneurons (red) in the somatosensory cortex. (**c**) Action potential firing pattern (top trace) elicited in a fast-spiking basket cell in response to a 1 s duration depolarizing current pulse (lower trace).

To determine whether PV interneuron circuits were affected by sensory deprivation, we used double transgenic mice expressing ChR2-eYFP specifically in PV-expressing interneurons (See Methods and ref. 25). PV neurons throughout the brain of these mice express ChR2-eYFP, most notably in cortex and cerebellum (Fig. 1a). Immunohistochemical imaging of ChR2-eYFP and PV expression in the barrel cortex showed that ChR2-eYFP eYFP was restricted to PV-positive interneurons (Fig. 1b).

We first examined our ability to photostimulate PV interneurons expressing ChR2-eYFP. Fast-spiking PV basket cells were identified based on several electrical criteria: membrane potentials of −78.6 mV ± 0.8 mV (n = 51) that were relatively depolarized compared to pyramidal neurons (−95.3 mV ± 0.5 mV), high-frequency action potential firing without adaptation in response to a depolarizing current pulse, and an undershoot following each action potential^34, 35^ (Fig. 1c). These are within the range of values previously reported for PV interneurons^35^ and pyramidal neurons^34–37^, after taking into account liquid junction potentials and specimen age. We did not observe any significant differences in the resting membrane potentials of these interneurons in deprived (−79.3 mV ± 0.7 mV) and control (−78 mV ± 1.2 mV) slices (p = 0.5, Mann-Whitney two-tailed test, n = 20 for control and n = 24 for deprived cells).

**Figure 2:**
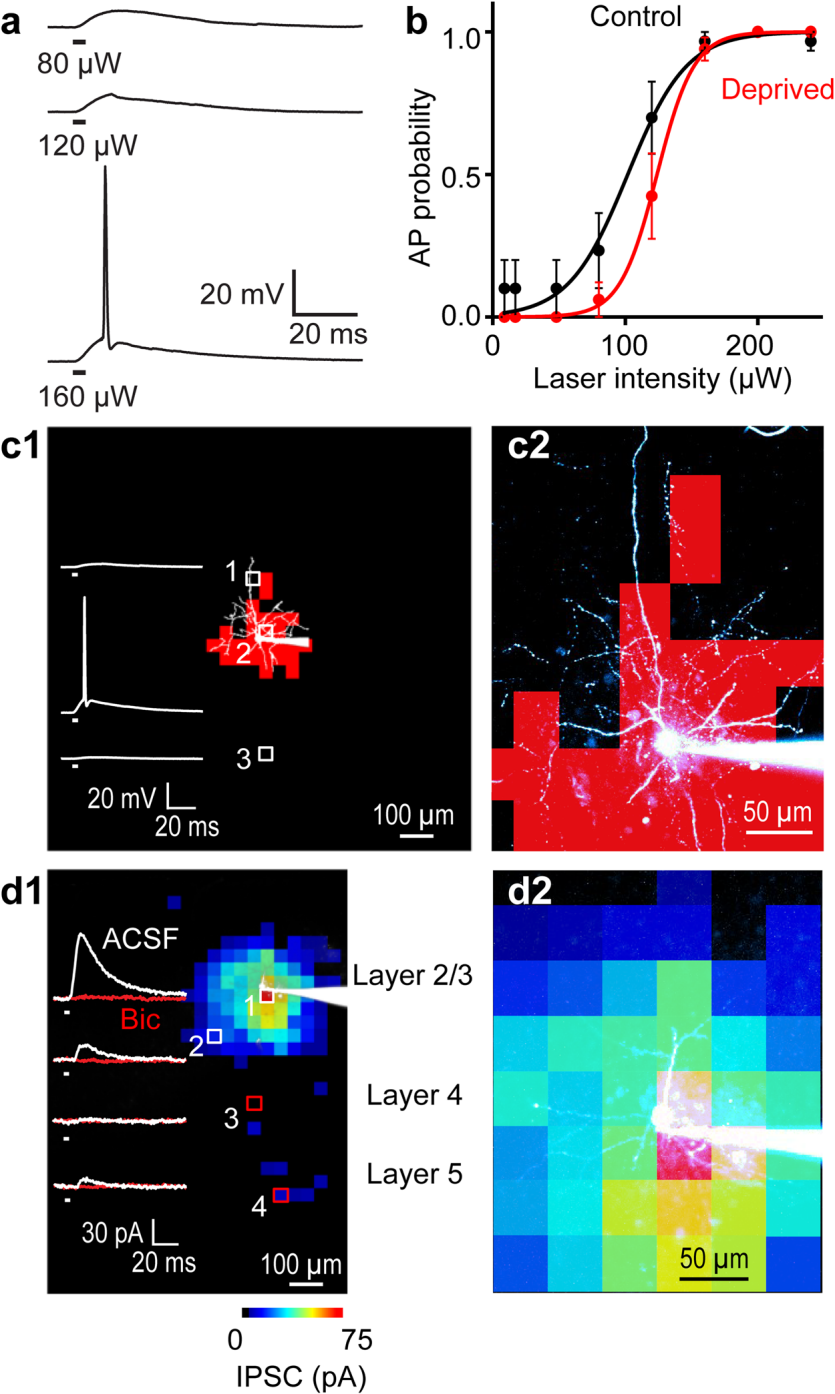
Parvalbumin-expressing interneurons can be reliably photostimulated. (**a**) Responses elicited in a PV cell interneuron by somatic photostimulation at different light intensities. Duration of photostimulation indicated by black bars under traces. (**b**) A cumulative probability histogram showing that 160 µW laser power was sufficient to reliably photostimulate ChR2-expressing PV interneurons. Error bars represent s.e.m. (c1) Left – Spatially resolved photostimulation of a PV interneuron (shown in white image). Traces illustrate voltage responses to numbered locations in interneuron image. Light spots near the cell body elicited action potentials (2), but not in locations at the ends of the proximal dendrites (1) or in another layer (3). Red pixels in interneuron image indicate laser locations that evoked action potentials (optical footprint). (c2) Right - Magnified version of left image, more clearly showing the position of the PV interneuron in the optical footprint map. (**d**) Mapping of inhibitory inputs onto postsynaptic layer 2/3 pyramidal neurons, with current traces shown on the left. (d1) Left - Inhibitory responses (white traces) were blocked by bicuculline (red traces). Map of spatial distribution of IPSCs is superimposed on image of pyramidal neuron, with IPSC amplitude encoded in pseudocolor scale shown below. (d2) Right - Magnified version of map and pyramidal cell image shows the relationship of inhibitory inputs to the pyramidal neuron. Strongest IPSC responses were evoked near the pyramidal cell soma.

Application of brief laser light spots (405 nm, 4 ms, approximately 2.1 μm^2^ area in the focal plane) caused the membrane potential of basket cells to depolarize, with the brightest pulses eliciting action potentials (Fig. 2a). Light flash intensity was varied to find an optimal stimulus for focal photostimulation: 160 µW flashes were able to elicit spiking reliably in basket cells from both control and deprived sides (Fig. 2a and b). Consistent with the observation that recombination in *Pvalb*-ires-Cre mice mostly occurs in large interneurons with the strongest PV expression^27^, photostimulation at such moderate laser intensities was able to evoke action potentials in fast-spiking basket cells but not in non-fast-spiking PV interneurons (data not shown). Similarly, no light-evoked depolarizations or inward photocurrents were measured in pyramidal neurons, indicating a lack of ChR2 expression in these cells as well.

We defined the spatial resolution of focal photostimulation by determining the “optical footprint” of these neurons, which is the area over which focal photostimulation generated action potentials^24^. By scanning the laser spot across the field of view in a pseudorandom fashion, to avoid sequential stimulation of adjacent pixels, we were able to identify and map the area of a ChR2-expressing PV interneuron that elicited action potential firing (red pixels in Fig. 2c1 and 2). Under our conditions, 160 µW flashes yielded median optical footprints of 31.4 × 10^3^ µm^2^ for control slices (n = 12 cells), roughly corresponding to a circle with a radius of 100 µm. The median optical footprint for deprived slices (26.9 × 10^3^ µm^2^; n = 13 cells) was not significantly different (p = 0.8, Mann-Whitney two-tailed test). These optical footprints are relatively large compared to those measured in cerebellar interneurons by Kim *et al*.^24^, but still yielded sufficient spatial resolution to differentiate between cortical layers and columns. The low resolution was caused, in part, by the low numerical aperture (0.3) of the microscope objective that we used.

By focally photostimulating presynaptic PV interneurons with the scanned laser spot while measuring light-evoked inhibitory postsynaptic currents (IPSCs) in layer 2/3 pyramidal cells, we could use our functional read-out to map the spatial organization of this inhibitory circuit (Fig. 2d1 and 2)^23, 24^. Because PV interneurons target pyramidal cell somata and proximal dendrites, these IPSCs were unlikely to be influenced by dendritic filtering^38^. Photostimulation over a broad field of view permitted mapping of PV interneuron connectivity across layers 1–6 and over 3–4 cortical columns. IPSCs occurred within the first 20 ms after the onset of a light flash and could be reliably evoked when photostimulating PV interneurons within layers 2/3 or 4; responses were sometimes evoked by photostimulation of layer 5 as well (Fig. 2d1). The range of light-evoked inhibitory input is consistent with the broad distribution of PV interneurons in the somatosensory cortex^12^ and with the ability of these neurons to target pyramidal neurons across layers. Application of bicuculline (10 µM), a competitive antagonist of GABA_A_ receptors, eliminated these responses and confirmed that they were IPSCs (Fig. 2d1).

**Figure 3:**
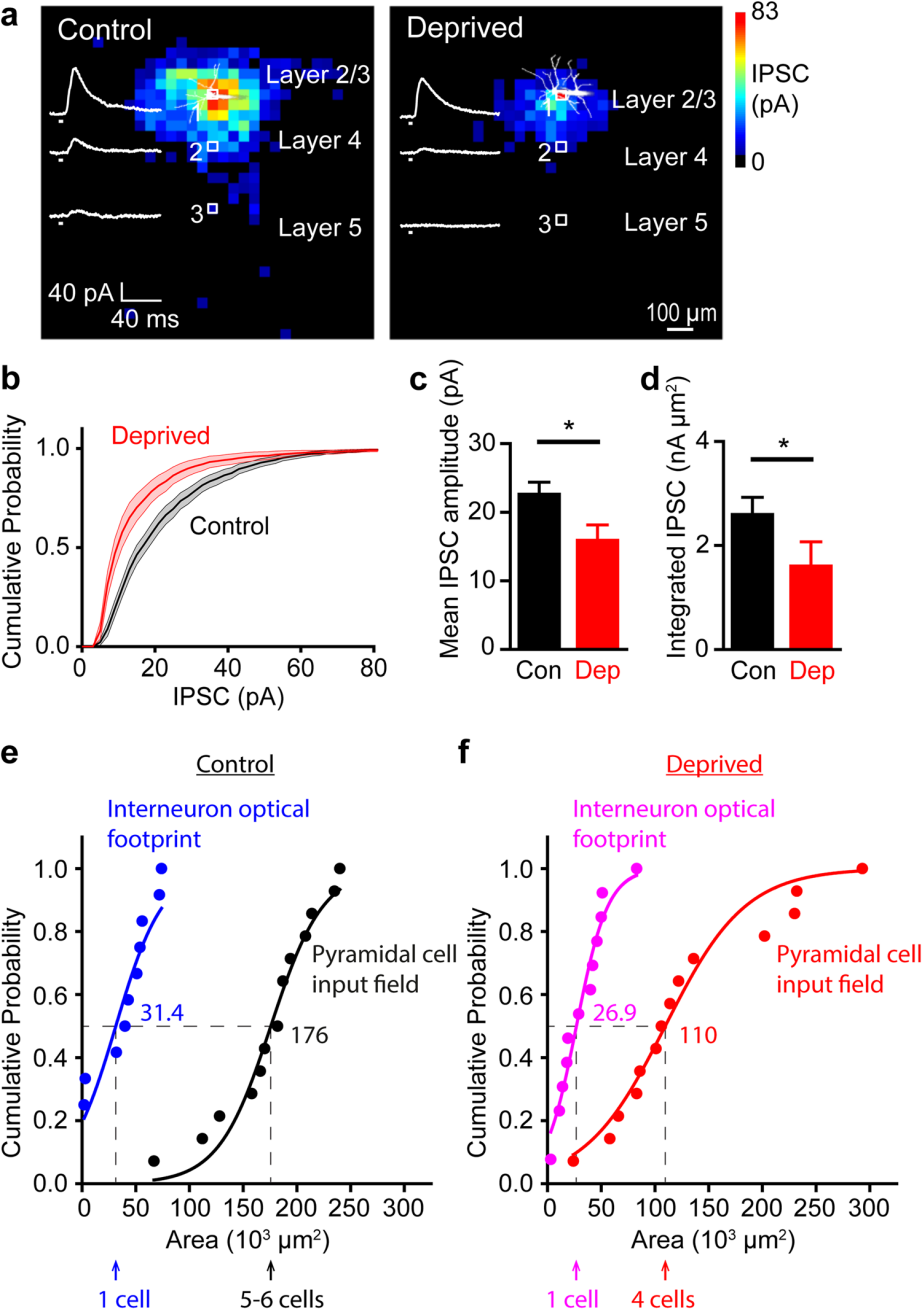
Chronic sensory deprivation decreased inhibitory transmission between PV interneurons and layer 2/3 pyramidal cells. (**a**) IPSC input maps of pyramidal cells in slices from control and deprived cortex from a mouse deprived at P0. Traces of illustrated IPSC responses recorded in response to numbered locations in layers 2/3, 4 and 5. (**b**) Cumulative probability distributions for IPSCs measured in response to PV interneuron photostimulation. IPSCs are smaller in deprived cortex (red). (**c**) Chronic deprivation decreased mean IPSC amplitudes (p = 0.016, Mann-Whitney two-tailed test, n = 14 cells each for deprived and controls, corresponding to 11 animals for deprived, 12 animals for controls). Error bars represent s.e.m. (**d**) Chronic deprivation decreased amplitude of integrated IPSC, measured as the sum of all IPSC responses above threshold. Treatment groups are significantly different (p = 0.019, Mann-Whitney two-tailed test, n = 14 cells each for deprived and controls, corresponding to 11 animals for deprived, 12 animals for controls). (**e**) Cumulative probability distributions for areas of optical footprint of control PV interneurons (blue) and IPSC input fields for control pyramidal neurons (black). The medians for the interneuron optical footprints and pyramidal cell IPSC input maps are indicated by dashed lines. Each data point represents a cell. (**f**) Cumulative probability distributions for areas of optical footprint of deprived PV interneurons (violet) and IPSC input fields for deprived pyramidal neurons (red). The medians for the interneuron optical footprints and pyramidal cell IPSC input maps are indicated by dashed lines. Each data point represents a cell.

To determine whether sensory activity controls the efficacy of inhibitory circuits, we compared the amplitude of IPSCs evoked in deprived and control slices (Fig. 3a). Chronic deprivation, beginning at P0, evoked smaller IPSCs (Fig. 3b). Mean IPSC amplitude was significantly reduced by deprivation (p = 0.016, Mann-Whitney two-tailed test, n = 14 cells each for deprived and controls, corresponding to 11 animals for deprived, 12 animals for control), as shown in Fig. 3c. To account for PV input being spatially distributed, IPSC amplitudes for each pixel within an input map were summed to measure the total inhibitory drive received by a pyramidal cell. This measure also was significantly reduced (p = 0.019) in deprived cortex as compared to controls (Fig. 3d). The decrease in inhibition could indicate decreases in circuit convergence, synaptic efficacy, or both.

### Sensory deprivation affects spatial organization of inhibitory circuits

Loss of sensory experience also affected the spatial organization of the local inhibitory circuit between PV interneurons and pyramidal cells. In control slices, input maps were large in area (176 ± 9 × 10^3^ µm^2^, n = 43 cells), while input maps in deprived slices were smaller (132 ± 21 × 10^3^ µm^2^, n = 12 cells). Two-sample Kolgomorov-Smirnov tests of cumulative input circuit areas showed deprivation significantly (p = 0.04) reduced the area over which pyramidal cells received input from PV interneurons (Fig. 3e and f). As mentioned previously, the area of PV interneuron optical footprints was not significantly affected by whisker deprivation (compare Fig. 3e and f); thus, the number of presynaptic PV interneurons converging onto a pyramidal cell should be proportional to the area of the IPSC input field. A lower estimate of the amount of convergence can be determined by dividing the median area of IPSC input fields by the median optical footprint area for PV interneurons^24^: deprivation decreased the apparent number of PV interneuron inputs to layer 2/3 pyramidal cells from a median of 5.6 inputs for controls to 4.1 inputs for deprived (Fig. 3e and f). This indicates that sensory deprivation decreases the number of inhibitory inputs provided to a pyramidal cell by PV interneurons; thus, sensory experience enhances formation of functional circuits between PV interneurons and pyramidal cells.

**Figure 4:**
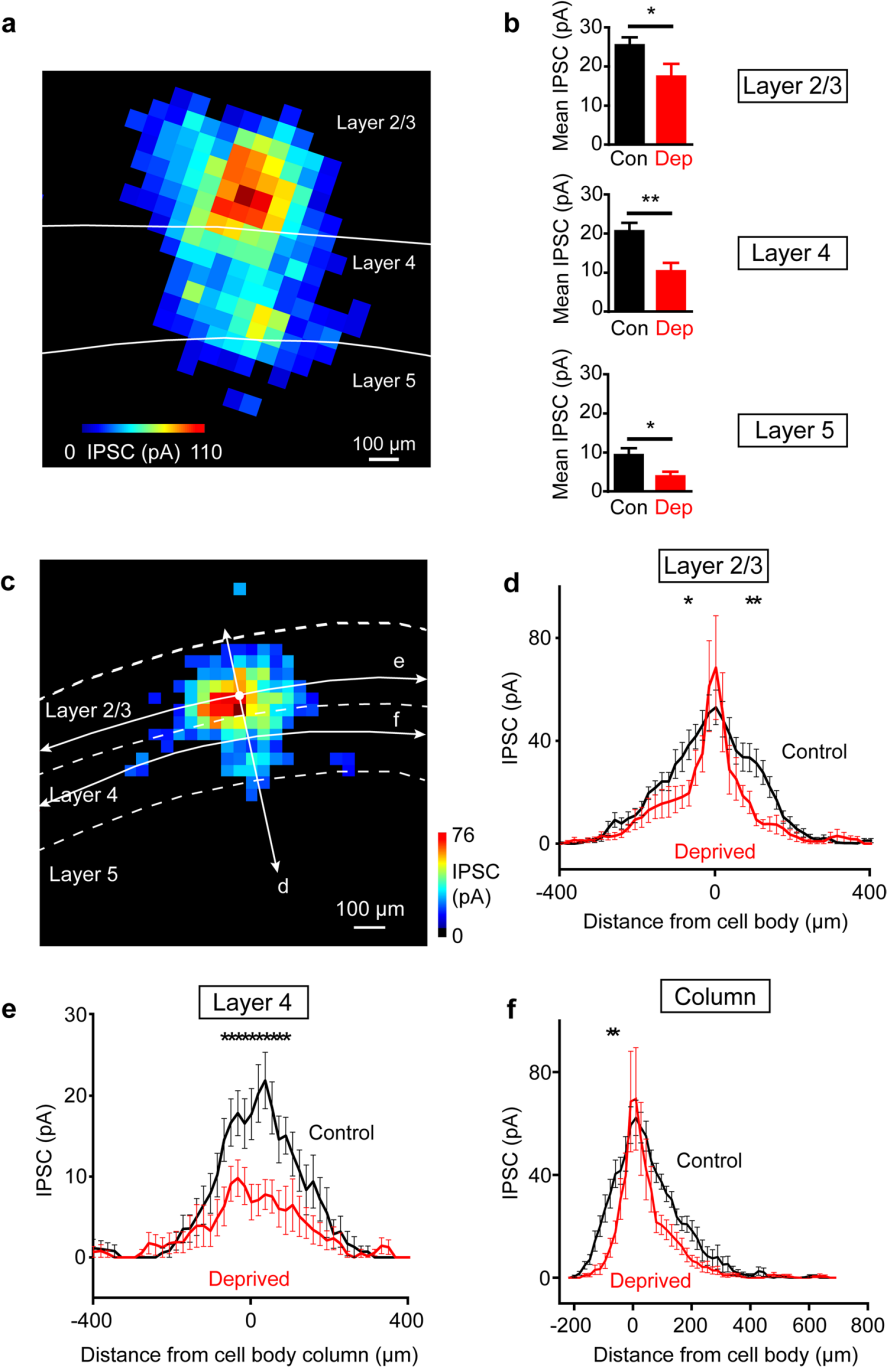
Chronic sensory deprivation decreased IPSC amplitudes across layers. (**a**) IPSC input map for a pyramidal cell in a control slice showing IPSC inputs from layers 2/3, 4 and 5. Layer location was determined from DIC images of slices. (**b**) Mean IPSC amplitudes were decreased for deprived slices across layers 2/3, 4 and 5. Error bars represent s.e.m. (**c**) IPSC map showing a line scan of responses along layers 2/3 and 4 (horizontal white arrows) and also along the column (vertical white arrow), with the position of the cell indicated by a white dot. (**d**) Averaged line scan of responses from IPSC maps showing that chronic deprivation narrowed the range of IPSC responses along layer 2/3 with the cell body as the reference point. Error bars represent s.e.m. (n = 14 cells each for controls and deprived, corresponding to 12 animals for control and 11 animals for deprived). Two-way ANOVA was done for the line scan with Bonferroni post-hoc tests indicating a significant effect of deprivation on IPSC responses p < 0.0001. (**e**) Averaged line scan of responses from IPSC maps showing that chronic deprivation also decreased the amplitude of IPSC responses along layer 4 with increasing distance from the center, with the position of the cell body column as the reference point. Error bars represent s.e.m. (n = 14 cells each for controls and deprived, corresponding to 12 animals for control and 11 animals for deprived). Two-way ANOVA was done for the line scan with Bonferroni post-hoc tests indicating a significant effect of deprivation on IPSC responses p < 0.0001. (**f**) Averaged line scan of responses from IPSC maps showing that chronic deprivation also decreased the amplitude of IPSC responses along the column with increasing distance from the center, with the position of the cell body as the reference point. Error bars represent s.e.m. (n = 14 cells each for controls and deprived, corresponding to 12 animals for control and 11 animals for deprived). Two-way ANOVA was done for the line scan with Bonferroni post-hoc tests indicating a significant effect of deprivation on IPSC responses p < 0.0001.

Maps of PV interneuron input onto layer 2/3 pyramidal neurons suggested that the effects of sensory deprivation were largest at the periphery of the input field, both within layer 2/3 and between layers (compare Fig. 3a left and right). We first compared the strength of PV interneuron circuits within each layer. In control conditions, IPSCs evoked in layer 2/3 pyramidal cells in response to photostimulation of layer 2/3 were largest and those in layer 5 were smallest (Fig. 4a and b). Deprivation significantly decreased the mean amplitude of IPSCs evoked in response to photostimulation of each of these layers (p = 0.023 for layers 2/3, 0.0016 for layer 4, and 0.034 for layer 5; Mann-Whitney two-tailed test; n = 14 cells each for deprived and controls, corresponding to 11 animals for deprived, 12 animals for controls). Collectively, these changes are sufficient to account for the net reduction in inhibitory drive caused by sensory deprivation (Fig. 3b–d). In addition, half of the layer 2/3 pyramidal cells that we recorded from in deprived slices did not receive PV interneuron input from layer 5, compared to approximately a fifth of the controls. While layer 2/3 PV interneurons do have axons that descend into layer 5^22^, the fact that our measured optical footprints of layer 2/3 PV cells did not extend into layer 5 (Fig. 2c1) indicates that these axons were not being photostimulated. Thus, the loss of inhibitory input when photostimulating layer 5 following sensory deprivation arises from a preferential reduction in longer-range connectivity between layer 5 PV interneurons and pyramidal cells.

Because PV inputs from different layers are differentially affected by sensory deprivation, we next examined how the distribution of inputs varied with distance. Inhibitory inputs within a layer typically spanned across 2–3 columns. Line scans of input maps, along layer 2/3 (arrows in Fig. 4c), showed that IPSCs evoked by photostimulating PV interneurons were largest nearest the pyramidal cell body and decreased with distance across the width of the columns (black plot in Fig. 4d). Whisker deprivation narrowed the spatial range of IPSC input along layer 2/3: IPSC amplitude was comparable to controls near the cell body, but decreased sharply with distance along layer 2/3 (red plot in Fig. 4d). This provides another indication of preferential loss of more distant PV interneuron inputs. Inhibitory inputs evoked along layer 4 decreased with distance across the width of the columns (black plot in Fig. 4e), similar to layer 2/3, though in this case the largest deprivation-induced reductions in IPSC amplitude were observed in the center of the inhibitory field (red plot in Fig. 4e). In addition, whisker deprivation narrowed the spatial range of IPSC inputs along the column, most noticeably in layers 2/3 and 4 (Fig. 4f). Collectively, these results indicate that sensory experience influences the spatial organization of local inhibitory circuits, preferentially affecting connections between more distant interneurons and their postsynaptic targets.

### Critical period for activity-dependent regulation of PV interneuron circuits

**Figure 5:**
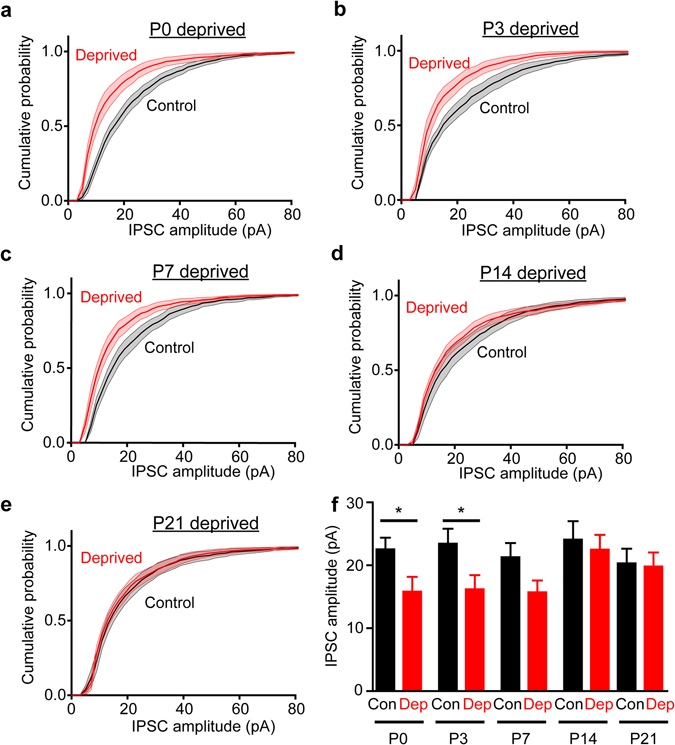
The sensitivity of PV interneuron-mediated IPSCs to whisker deprivation decreases with developmental age. (**a**)–(**e**) Averaged probability cumulative distributions of IPSCs for deprived slices converged with controls at P14 and P21 deprivation. (**f**) Mean inhibition in deprived slices was decreased significantly with deprivation starting at P0 (p = 0.016) and P3 (p = 0.032), and recovered to similar levels to controls when the deprivation date was delayed to P7, P14 and P21 (p = 0.073 at P7, 0.48 at P14, and 0.80 at P21 respectively; Mann-Whitney two-tailed test comparing only within each timepoint group independently, n numbers of P0: 14 cells each for controls and deprived, corresponding to 12 animals for controls, 11 animals for deprived, P3: 9 cells each for controls and deprived, corresponding to 6 animals each for controls and deprived, P7: 9 cells controls and 14 cells deprived, corresponding to 8 animals for controls, 12 animals for deprived, P14: 8 cells controls and 11 cells deprived, corresponding to 5 animals for control, 7 animals for deprived, P21: 12 cells each for controls and deprived, corresponding to 9 animals for control, 7 animals for deprived). Error bars represent s.e.m.

The time course of critical periods is defined by determining the time period over which sensory deprivation induces changes in cortical responsiveness^1^. We determined the critical period for sensory experience to influence PV interneuron circuits by varying the time at which whiskers were removed, with deprivation dates of P0, P3, P7, P14 and P21, while measuring effects on inhibitory circuits at P30 in all cases. In control slices, IPSCs evoked by photostimulation of PV interneurons had a mean amplitude of 23.6 pA ± 0.4 pA (Fig. 5a) and were relatively consistent across all five control groups (Fig. 5a–e). While whisker deprivation at early times influenced PV interneuron inhibitory circuits, deprivation of whisker activity at later times had progressively less effect on inhibition (Fig. 5). IPSCs were smaller than controls when deprivation was initiated at P0 or P3 (Fig. 5a and b), but this difference between control and deprived slices was reduced when deprivation was delayed to P7 (Fig. 5c), and the two distributions converged when deprivation started at P14 (Fig. 5d) or at P21 (Fig. 5e). While mean IPSC amplitudes were significantly different from controls when deprivation was started at P0 (p = 0.016; Mann-Whitney two-tailed test) and P3 (p = 0.032) (Fig. 5f), there were no differences in mean IPSC amplitude when deprivation was done at P7 (p = 0.073), P14 (p = 0.48) or P21 (p = 0.80). Means were similar when calculated across animals instead of the number of cells (data not shown). These results indicate a sharp, well-defined critical period in which sensory experience influences development of the PV interneuron-pyramidal cell inhibitory circuit.

**Figure 6:**
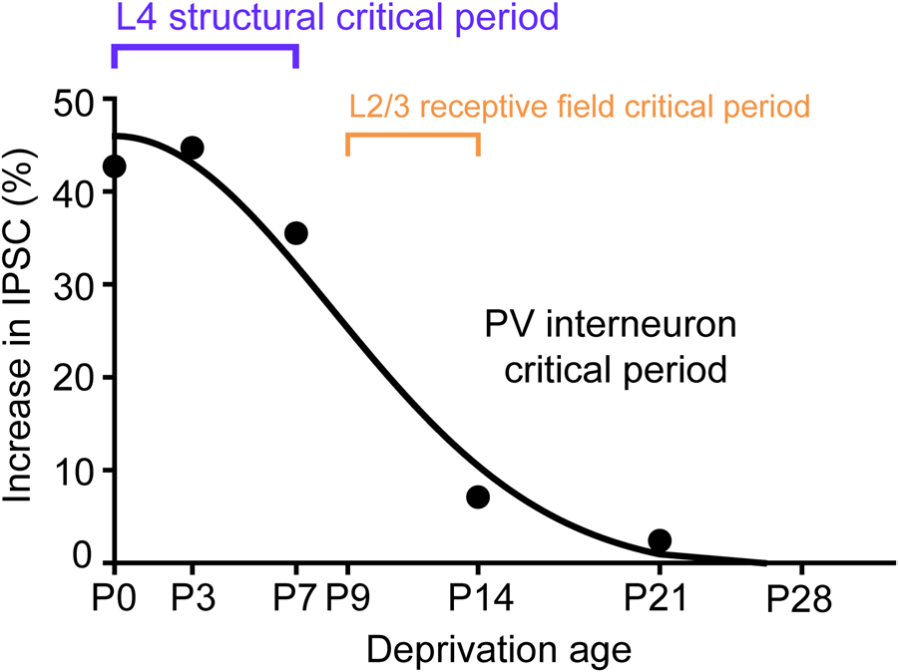
A critical period for experience-dependent plasticity in the inhibitory circuit. Curve depicts differences in mean IPSCs measured between control and deprived slices at different times of starting whisker deprivation. Sensory experience increases IPSCs in controls relative to deprived slices. The largest experience-dependent changes in IPSCs occurred at P0 and P7, decreasing sharply at P14 and P21 time points. Curve is a half-Gaussian fit with a half-width of approximately 10 days (R^2^ = 0.95). Critical periods observed in layers 2/3 and 4 in the somatosensory cortex. The layer 2/3 receptive field critical period (yellow) was assembled from observations reported previously^40–42^. The layer 4 critical period (blue) illustrates the relationship between layer 4 barrel size and deprivation start date^39^.

To quantify the critical period for this inhibitory circuit, we calculated the difference between control and deprived slices at different time points of deprivation. The effect of experience on IPSC amplitude was maximal at P0 and decreased when whisker input was deprived at later ages (Fig. 6). This relationship could be fit (R^2^ = 0.95) with a half-Gaussian function with a width (at half-maximum) of approximately 10 days. Very similar results were observed when comparing the distances between the averaged IPSC distributions (vertical distance calculated with the Kolgomorov-Smirnov test) for deprived and control samples (data not shown). In conclusion, the sensitivity of PV interneuron inhibitory circuits to whisker deprivation is high at birth and decreases sharply afterwards, being insensitive to sensory input by P21 (Fig. 6). Thus, sensory input normally strengthens the circuit between PV interneurons and pyramidal cells over the first two weeks of postnatal development and has little or no effect for the remainder of postnatal development.

## Discussion

While the critical periods for barrel structure^6, 39^ and for excitatory circuits in layers 4 and 2/3 of the barrel cortex^40–43^ have been well-characterized, there are no previous functional analyses of the time course of experience-dependent plasticity in any cortical inhibitory circuit. Hence, the extent to which these circuits are regulated by sensory experience was unknown; it also was not clear whether the development of inhibitory circuits onto layer 2/3 pyramidal neurons exhibits a defined critical period. We answered these questions by using optogenetics to selectively interrogate PV interneurons and to map the spatial organization of inhibitory circuits between these neurons and their layer 2/3 pyramidal neuron targets at different developmental time points.

Our results provide several lines of evidence that inhibitory circuits in the somatosensory cortex of mice undergo activity-dependent changes during a well-defined critical period. First, we found that chronic whisker deprivation decreased inhibition of layer 2/3 pyramidal cells mediated by PV interneurons, specifically basket cells. Secondly, this decrease in the strength of inhibition was observed for presynaptic PV interneurons located in layers 2/3, 4 and 5. Notably, deprivation resulted in a distance-dependent scaling of inhibition, with inputs farthest from the postsynaptic pyramidal cells preferentially reduced. As a result, connectivity between layer 5 PV interneurons and layer 2/3 pyramidal cells was nearly eliminated by deprivation. Lastly, we observed a critical period for this plasticity: sensitivity to whisker experience was reduced by half approximately 10 days after birth and is completely absent 3 weeks after birth.

In our study, unilateral whisker deprivation was used: deprived (experimental) and undeprived (control) somatosensory cortices were then compared, in the same animal, to determine the effects of loss of sensory experience. When examining the effects of sensory deprivation on the visual cortex, the undeprived side also is often used as a control^44–46^. This paradigm could cause interhemispheric effects on the control side, particularly via the influence of long-range callosal connections^47^. However, this paradigm does avoid many potential competitive effects on local cortical circuits resulting from partial whisker deprivation^28, 48, 49^ and, thus, seems most appropriate for examining the effects of sensory deprivation on local inhibitory circuitry. Formally speaking, our results describe the relative changes between deprived and undeprived hemispheres, rather than the absolute effect of the loss of activity on the deprived hemisphere.

### Mechanisms underlying the critical period for interneuron circuits

In control conditions, PV interneuron input to layer 2/3 pyramidal cells was broadly distributed and appeared similar to observations made by photostimulating all *Gad2*-expressing interneurons^50^. Whisker trimming from P9 to P14 reportedly does not induce significant changes in layer 2/3 inhibitory input, as determined by glutamate uncaging mapping at P14–16^41^. This is consistent with our observation that deprivation at P14 has minimal effect on PV interneuron circuits (Fig. 6). Photostimulation of other types of interneurons during glutamate uncaging might mask the substantial effects on PV interneurons that we would predict at day P9.

Chronic deprivation from P0 reduced the strength and spatial range of the inhibitory circuits between presynaptic PV neurons and their postsynaptic layer 2/3 pyramidal cell targets (Fig. 3). In principle, the reduction in IPSC amplitude could result from a decrease in the convergence of PV interneuron inputs onto pyramidal cells and/or a reduction in the strength of these synaptic connections. We observed a decrease in the degree of apparent convergence, which declined from 5–6 to 4 presynaptic PV cell inputs (Fig. 3e and f). Because our focal photostimuli were likely to activate a small number of basket cells, it is unclear whether there are also activity-dependent effects on synaptic efficacy.

The reduction in apparent synaptic convergence that we observed is consistent with a report that P7 deprivation decreases the number of PV-positive interneurons innervating layer 4 spiny neurons, as well as reducing the number of synapses from PV-expressing basket cells and IPSCs (evoked by stimulating undefined interneurons)^12^. Our observations show that the circuits formed between PV interneurons and their layer 2/3 targets respond similarly to those interneuron circuits innervating layer 4^12^ and further show that sensory deprivation changes the spatial organization of PV interneuron local circuits (Fig. 4). Whisker deprivation narrowed the spatial range of IPSC inputs to layer 2/3 pyramidal neurons along layer 2/3 and along the column (Fig. 4d). The narrowing of inhibition on layer 2/3 pyramidal neurons would be expected to increase spread of excitation along the layer and is consistent with reports that sensory deprivation broadens layer 2/3 cell receptive fields^42^. Surprisingly, changes in the distribution of layer 4 PV cell inputs to layer 2/3 pyramidal cells were largest in the center of the inhibitory field (Fig. 4e). While layer 4 PV interneurons target both layer 2/3 pyramidal neurons and layer 4 spiny neurons, receptive field maps in layer 4 are stable during whisker deprivation^42^. Hence, sensory deprivation effects on PV circuits might have a larger net effect on regulating inhibition of postsynaptic layer 2/3 pyramidal neurons compared to layer 4 spiny neurons.

### Defining the critical period of an inhibitory circuit

By determining the sensitivity of the circuit between PV interneurons and layer 2/3 pyramidal neurons to sensory stimuli at different ages, we discovered that the critical period for PV interneuron synaptic plasticity spans P0–P14 (Fig. 6). Our characterization of the PV interneuron critical period can serve as a benchmark for identifying possible molecular mediators of critical period plasticity in this circuit^51^. In order to define this relationship, we varied the sensory deprivation start time while fixing the recording endpoint of the experiment, similar to the method previously used to define the critical period for barrel formation^39^. This paradigm also facilitated comparison across experimental groups by ensuring that the cortical circuits were of similar maturity and that channelrhodopsin expression, which varies with age, was constant at the time of mapping.

Our results indicate that sensory deprivation after P21 does not affect the circuit between PV interneurons and pyramidal neurons (Fig. 6). When combined with a recent study reporting that deprivation in adulthood (around 8–11 weeks) also does not change the distribution of PV interneurons in layers 4 and 5 or alter IPSCs mediated by *Gad2* ^*+*^ interneurons in layer 2/3^14^, we can conclude that layer 2/3 PV circuit plasticity is restricted to the age range defined by our results (Fig. 6). An alternative explanation for the lack of change in PV inputs when deprivation was done at later times could be the resulting shorter duration of deprivation. While it is possible that experience-dependent plasticity of PV interneurons requires a defined duration of deprivation, rather than having a defined critical period, this is unlikely because another study indicated that whisker deprivation up to 3 weeks past the critical period does not alter layer 2/3 IPSCs^14^. Hence, the time of deprivation relative to the critical period seems more important than the absolute duration of whisker deprivation.

Different inhibitory circuits have different responses to sensory deprivation. While we observed that the distribution of PV interneuron inputs - as well as horizontal connections between layer 2/3 pyramidal neurons and interneurons in all layers - does not change once the postnatal critical period has passed, removing whisker input in adulthood (8–11 weeks) produces a transient decrease in inhibition ascending from layer 5 to layer 2/3^14^. This effect is likely mediated by Martinotti interneurons, which appear to be more plastic and can serve as targets of transient activity-dependent plasticity even in adulthood. Even more remarkable is the bidirectional effect of activity on inhibitory inputs onto pyramidal neurons that has been observed in layer 4 of the visual cortex^52^, which contrasts with the exclusively positive regulatory influence of activity we observed for the PV interneuron inputs to pyramidal cells in layer 2/3 of somatosensory cortex (Fig. 6). Apparently different rules apply to different types of cortical inhibitory circuits during the critical period.

Likewise, multiple critical periods exist for excitatory circuits in different layers of the barrel cortex^53^; even within layer 2/3, discrete critical periods can be observed for intralaminar and interlaminar excitatory synapses^43^. These critical period processes span different time ranges during brain development and are associated with a range of structural or physiological changes in individual circuits^53–55^. The existence of multiple critical periods and types of plasticity apparently reflects differential activity-dependent development of individual circuits within the barrel cortex.

### Functional significance of interneuron circuit critical period plasticity

The P0–P10 critical period window that we identified for the circuit between PV interneurons and layer 2/3 pyramidal neurons overlaps with the critical period of the layer 2/3 receptive field: in rats, the stable organization of L2/3 receptive fields emerges at P14, while sensory deprivation before P14 disrupts receptive field structure in layer 2/3^42^ (Fig. 6). The P0–P10 critical period window for PV inhibitory circuits also coincides with a rapid increase in the density of cortical synaptic circuits^56^ and the onset of active exploratory whisking behavior^57^ at P10–P15. Previous studies on layer 2/3 plasticity described changes in excitatory circuits following sensory deprivation^40–43^. These correlations suggest that the experience-dependent increase in inhibition we observed could be involved in the development of excitatory circuits. Indeed, decreased inhibition in layers 2/3 and 4 caused by whisker deprivation (Fig. 4) could at least partially explain deprivation-induced changes in layer 2/3 receptive fields^42^. Sensory deprivation from P8 decreases excitatory synaptic transmission from layer 4 to layer 2/3, but does not affect axonal topography within these excitatory circuits^58^. Thus, the deprivation-induced broadening of layer 2/3 receptive fields observed by Stern *et al*.^42^ might be facilitated by the decrease in PV interneuron inhibition we have observed, with disinhibited layer 2/3 excitatory circuits contributing to (or even causing) larger receptive fields.

A gradual developmental increase in inhibitory circuit strength helps create the critical period in the visual cortex by allowing cortical plasticity when the inhibitory-excitatory balance is within an optimal range^59^. Because whisker activity regulates PV interneuron-mediated inhibition on layer 2/3 pyramidal neurons in somatosensory cortex (Fig. 6), it is possible that experience-dependent development of PV-mediated inhibition may similarly trigger the onset or closure of the layer 2/3 receptive field critical period. Having characterized a precise functional time course of an inhibitory critical period, it will be important to elucidate how experience-dependent plasticity occurs in different neuronal elements in the cortex and to better understand the role of PV interneuron plasticity in regulating such processes.
